# 637. Effect of Liberalized Daptomycin and Linezolid Use on Resistance in Staphylococcus and Enterococcus Isolates

**DOI:** 10.1093/ofid/ofaf695.201

**Published:** 2026-01-11

**Authors:** Blake A Jennewein, Karrine Brade, Meghan N Jeffres, Douglas N Fish

**Affiliations:** Carilion Clinic, Roanoke, Virginia; University of Colorado Hospital, Aurora, Colorado; University of Colorado Anschutz Medical Campus, Aurora, CO; University of Colorado Skaggs School of Pharmacy and Pharmaceutical Sciences, Aurora, Colorado

## Abstract

**Background:**

Compared to vancomycin, daptomycin and linezolid have more favorable safety and efficacy profiles and require less therapeutic monitoring. To capitalize on these advantages, the University of Colorado Hospital relaxed prescribing restrictions and updated local practice guidance for treatment of infections caused by Gram-positive pathogens, resulting in increased daptomycin and linezolid use. The objective of this study is to assess the relationship between increased consumption of daptomycin and linezolid and subsequent changes in minimum inhibitory concentrations (MICs) of Gram-positive organisms.

**Figure:**
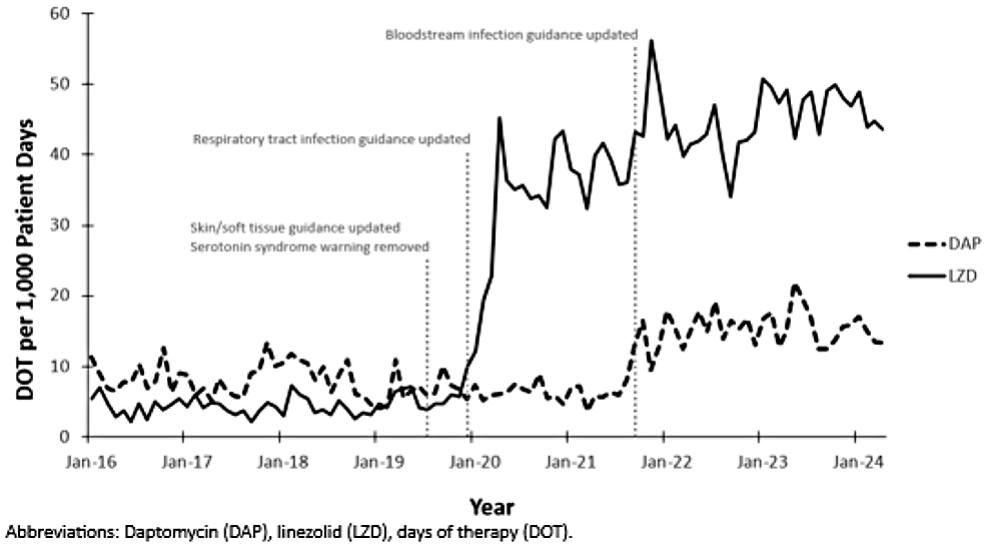
Monthly Daptomycin and Linezolid Usage Between January 2016 and April 2024

**Figure:**
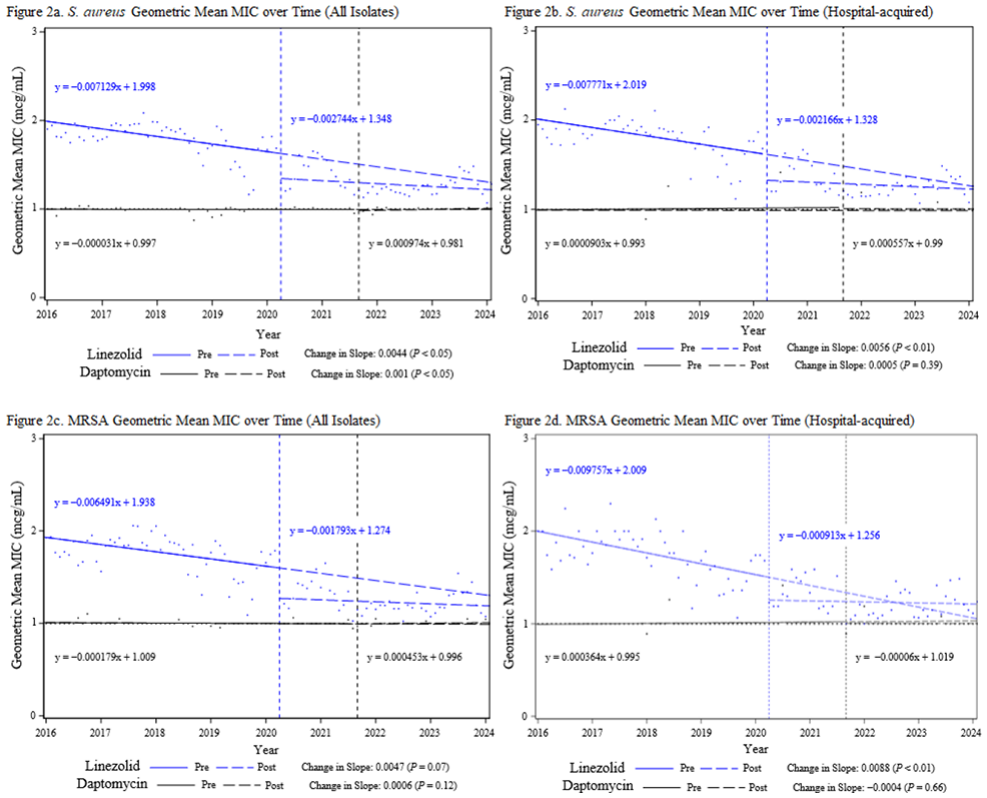
Staphylococcus aureus Geometric Mean MIC over Time

**Methods:**

This was a retrospective, single-center study of *Staphylococcus* and *Enterococcus* isolates collected across an 8-year period, including a 3-year follow-up period after updating local practice guidance. Organisms were further grouped as hospital-acquired if the culture was collected at least 48 hours after admission. Interrupted time series analyses were conducted to assess changes in MICs before and after implementation of practice updates related to daptomycin and linezolid prescribing.

**Figure:**
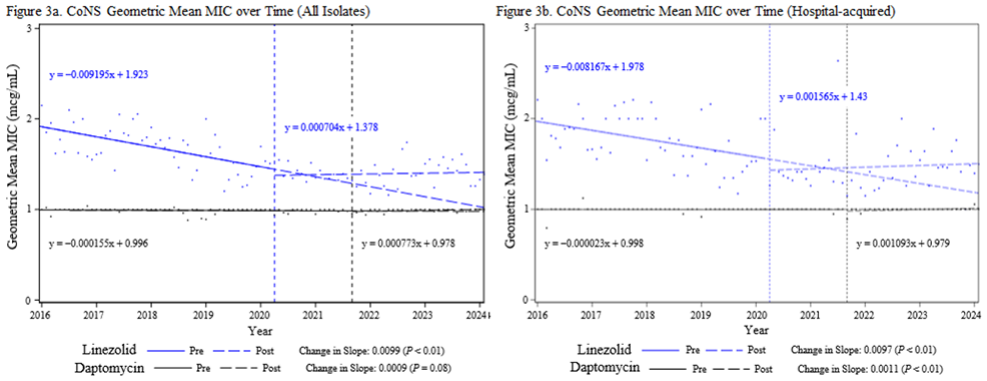
Coagulase-negative Staphylococcus Geometric Mean MIC over Time

**Figure:**
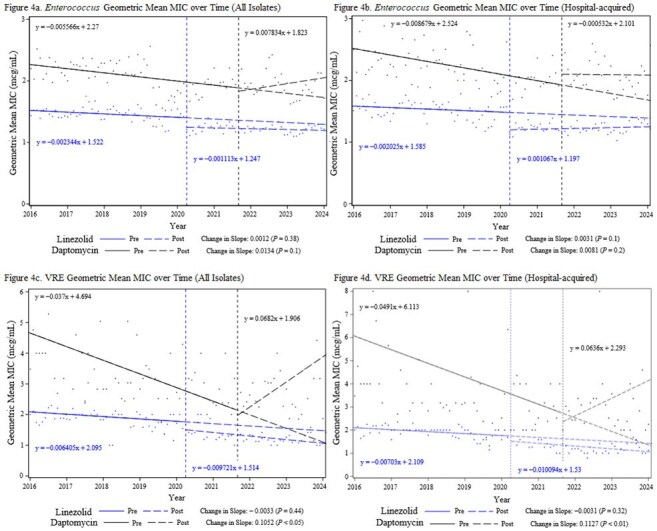
Enterococcus Geometric Mean MIC over Time

**Results:**

After implementation of local practice changes, linezolid usage increased from a baseline of 4.6 to 43.7 days of therapy per 1,000 patient days (DOT). Daptomycin use increased from a baseline of 7.6 to 15.7 DOT. Data from 19,160 isolates were reviewed, including 7,224 hospital-acquired isolates. Statistical analysis did not identify any increases in *Staphylococcus aureus* MICs post-implementation. In hospital-acquired coagulase-negative *Staphylococcus* (CoNS), MICs increased post-implementation for both daptomycin (*P* = .008) and linezolid (*P* = .002). In hospital-acquired vancomycin-resistant enterococci (VRE), only daptomycin MICs increased significantly post-implementation (*P* = .009). No clinically relevant increases in overall *Staphylococcus* or *Enterococcus* resistance rates were observed during the study period.

**Conclusion:**

The liberalization of daptomycin and linezolid use did not result in clinically relevant increases in MICs within the 3-year follow-up period. Further studies are needed to confirm the durability of this antibiotic use practice, specifically among CoNS and VRE.

**Disclosures:**

All Authors: No reported disclosures

